# Systemic Outcomes of (Pyr^1^)-Apelin-13 Infusion at Mid-Late Pregnancy in a Rat Model with Preeclamptic Features

**DOI:** 10.1038/s41598-019-44971-0

**Published:** 2019-06-12

**Authors:** Liliya M. Yamaleyeva, K. Bridget Brosnihan, Ebrahim Elsangeedy, Carolynne McGee, Sara Shi, David Caudell, Cynthia Miller, Jasmina Varagic, Michael Bader, Ralf Dechend, Hossam A. Shaltout

**Affiliations:** 1 Department of Surgery/Hypertension and Vascular Research, Wake Forest School of Medicine, Winston-Salem, North Carolina, United States; 2 Department of Obstetrics and Gynecology, Wake Forest School of Medicine, Winston-Salem, North Carolina, United States; 3 Department of Pathology, Section of Comparative Medicine, Wake Forest School of Medicine, Winston-Salem, North Carolina, United States; 4 Department of Pharmacology and Toxicology, School of Pharmacy, University of Alexandria, Alexandria, Egypt; 5 Max Delbrück Center for Molecular Medicine, Berlin, Germany; 6 Charité University Hospital Berlin, Berlin, Germany

**Keywords:** Reproductive biology, Experimental models of disease

## Abstract

Preeclampsia is a syndrome with diverse clinical presentation that currently has no cure. The apelin receptor system is a pleiotropic pathway with a potential for therapeutic targeting in preeclampsia. We established the systemic outcomes of (Pyr^1^)-apelin-13 administration in rats with preeclamptic features (TGA-PE, female transgenic for human angiotensinogen mated to male transgenic for human renin). (Pyr^1^)-apelin-13 (2 mg/kg/day) or saline was infused in TGA-PE rats via osmotic minipumps starting at day 13 of gestation (GD). At GD20, TGA-PE rats had higher blood pressure, proteinuria, lower maternal and pup weights, lower pup number, renal injury, and a larger heart compared to a control group (pregnant Sprague-Dawley rats administered vehicle). (Pyr^1^)-apelin-13 did not affect maternal or fetal weights in TGA-PE. The administration of (Pyr^1^)-apelin-13 reduced blood pressure, and normalized heart rate variability and baroreflex sensitivity in TGA-PE rats compared to controls. (Pyr^1^)-apelin-13 increased ejection fraction in TGA-PE rats. (Pyr^1^)-apelin-13 normalized proteinuria in association with lower renal cortical collagen deposition, improved renal pathology and lower immunostaining of oxidative stress markers (4-HNE and NOX-4) in TGA-PE. This study demonstrates improved hemodynamic responses and renal injury without fetal toxicity following apelin administration suggesting a role for apelin in the regulation of maternal outcomes in preeclampsia.

## Introduction

Preeclampsia is a multisystem syndrome characterized by a new onset hypertension and end-organ dysfunction with or without proteinuria^1^. It is associated with high maternal morbidity, premature birth, and intrauterine growth restriction. Women and children exposed to preeclampsia are more likely to develop cardiovascular disease later in life^2^. The prevalence and incidence of preeclampsia continue to increase in the United States^3,4^. However the pathogenesis of preeclampsia is still not well understood and treatment options remain very limited.

Apelin is a novel peptide with demonstrated beneficial cardiovascular effects in animal and human studies. Apelin exerts blood-pressure-lowering^5–8^, inotropic^9^, anti-apoptotic^10^, and pro-angiogenic effects^11^. Apelin acts on a G-protein-coupled receptor, apelin receptor (APJ). Several peptides are formed from the apelin precursor, and all apelin forms exert their effects through APJ signaling. Furthermore, pyroglutamate apelin-13 ((Pyr^1^)-apelin-13) is a posttranslationally modified form of apelin-13 containing the pyroglutamate group at the NH2 terminus of the peptide. We and others have demonstrated that apelin and APJ are expressed in human normal and preeclamptic placenta in syncytiotrophoblasts, cytotrophoblasts, and endothelial cells of fetal capillaries^12–14^. Apelin and APJ have similar distribution in human placenta, suggesting a paracrine effect of apelin receptor system. Using a quantitative radioimmunoassay, we have previously reported that total apelin content is lower in human preeclamptic chorionic villi suggesting reduced influence on regulation of the fetal-maternal interface^12^. We also demonstrated a complex profile and different patterns of apelin forms in human preeclamptic versus normal chorionic villi^12^. However, circulating levels of apelin were lower^15^, not different^16^, or higher^17^ in women with preeclampsia compared with women with normal pregnancy, probably reflecting different patient populations and assay conditions. Importantly, levels of apelin in maternal circulation correlate with levels of apelin in fetal plasma^18^ suggesting a reciprocal regulation of this peptide in mother and baby. In short, the levels and the distribution patterns of apelin and its receptor in human placenta suggest a potential of this peptide for therapeutic targeting in preeclampsia.

Since the systemic effects of exogenously administered apelin in preeclampsia are not well described, the goal of this study was to determine the effects of a systemically administrated and more stable form of apelin, (Pyr^1^)-apelin-13, on maternal and fetal characteristics, and cardiovascular and renal outcomes in an established rat model with preeclamptic features (TGA-PE, female transgenic for human angiotensinogen mated to male transgenic for human renin). This model was chosen based on the following characteristics: lower circulating levels of apelin, the expression of many features of human preeclampsia such as increased blood pressure at late pregnancy, intrauterine growth restriction, proteinuria, increased circulating sFlt and vasopressin, the presence of circulating agonistic antibodies to angiotensin II receptor 1, and glomerular endotheliosis versus Sprague Dawley rats (controls), and good reproducibility^19–21^.

## Results

### Maternal and Fetal Characteristics in TGA-PE versus SD rat

Table 1 shows that at the GD20, TGA-PE rats had lower maternal and pup weight, and number of pups compared with SD rats. There were no differences in placental weight or fetal-to-placental weight ratio. There were two pup resorptions noted in TGA-PE rats versus none noted in SD groups. In addition, heart-to-maternal body weight was higher in TGA-PE rats compared with controls. Water intake increased in TGA-PE versus SD females as did urine volume, but the differences were not statistically significant. There were no alterations in food intake.

**Table 1:** Maternal and Fetal Characteristics in Pregnant Sprague-Dawley (SD) and Transgenic Rats with Preeclamptic Features (TGA-PE) at Day 20 of Gestation.

	SD	SD + (Pyr^1^)	TGA-PE	TGA-PE + (Pyr^1^)
Maternal Weight, g	0.348 ± 0.01	0.377 ± 0.006	0.303 ± 0.009*	0.301 ± 0.007*
Pup Weight, g	3.68 ± 0.1	3.85 ± 0.11	3.21 ± 0.1*	3.17 ± 0.1*
Pup Length, cm	3.5 ± 0.03	3.7 ± 0.09	3.1 ± 0.15*	3.2 ± 0.09^
Number of Pups	14.5 ± 0.8	14.5 ± 0.6	10.7 ± 1.1*	12.7 ± 0.5
Placental Weight, g	0.45 ± 0.01	0.54 ± 0.02	0.41 ± 0.02	0.42 ± 0.01
Fetal/Placental Weight Ratio	8.10 ± 0.25	7.26 ± 0.31	7.90 ± 0.27	7.80 ± 0.28
Pup Resorption, Number	0	0	2	0
Maternal Total Kidney Weight/Body Weight, g	0.85 ± 0.03	0.95 ± 0.02	0.84 ± 0.02	0.84 ± 0.02
Maternal Heart Weight/Body Weight, g	0.80 ± 0.02	0.74 ± 0.01	1.17 ± 0.11*	1.04 ± 0.02
Food Intake, g	20.13 ± 2.2	20.5 ± 0.54	18.5 ± 1.55	16.45 ± 2.56
Water Intake, ml	37.11 ± 4.48	64.26 ± 15.96	55.47 ± 6.64*	45.45 ± 7.30
Urine Volume, ml	13.53 ± 2.25	7.66 ± 0.59	26.22 ± 6.25	37.52 ± 9.17*^
Serum Total Apelin Levels, ng/ml	1.6 ± 0.1	2.4 ± 0.1*	1.2 ± 0.10*	1.7 ± 0.10^#^^

Data are mean ± SEM, *p < 0.05 vs. SD; ^#^p < 0.5 vs. TGA-PE, ^p < 0.05 vs. SD-(Pyr^1^), n = 5–8.

### Effects of (Pyr^1^)-apelin-13 on Maternal and Fetal Characteristics, Blood Pressure, and Sympathovagal Activity

**Figure 1:**
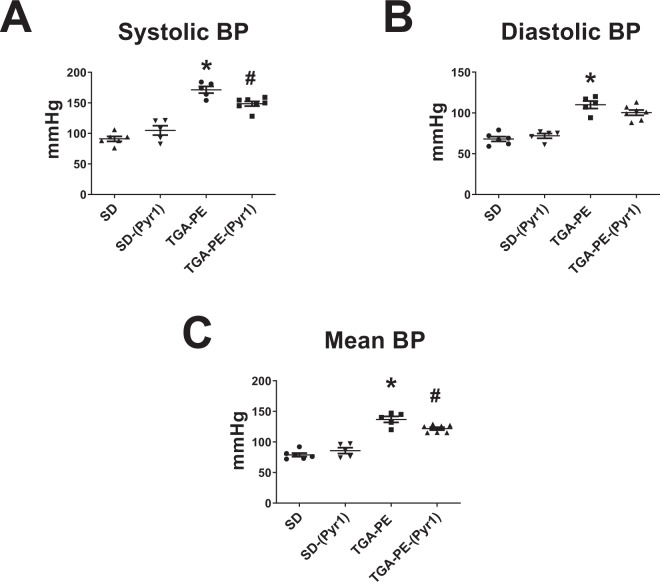
The effects of (Pyr^1^)-apelin-13 on systolic (**A**), diastolic (**B**), and mean (**C**) blood pressures in TGA-PE and SD rats at day 20 of gestation. Data are mean ± SEM. *p < 0.05 vs. SD saline-treated; ^#^p < 0.05 vs. TGA-PE saline-treated; n = 5–7.

**Figure 2:**
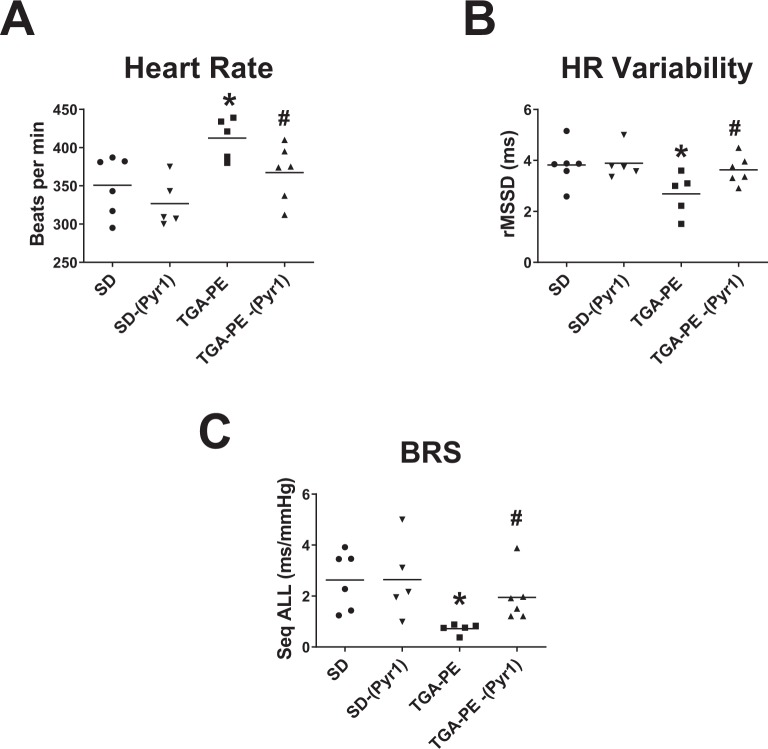
The effects of (Pyr^1^)-apelin-13 on heart rate (**A**), heart rate variability (**B**), and baroreflex sensitivity (**C**) in TGA-PE rats at day 20 of gestation. Data are mean ± SEM. *p < 0.05 vs. SD saline-treated; ^#^*p* < 0.05 vs. TGA-PE saline-treated; n = 5–7. Abbreviations: HR, heart rate; BRS, baroreflex sensitivity.

Serum levels of apelin were lower in TGA-PE compared with SD rats. Apelin infusion increased serum apelin in SD and TGA-PE rats compared with respective vehicle controls (Table 1). (Pyr^1^)-apelin-13 had no effect on maternal or fetal weight, pup number or length, or placental weight in TGA-PE or SD compared with respective controls. TGA-PE rats had higher mean and systolic blood pressures compared with SD rats **(**Fig. 1**)**. (Pyr^1^)-apelin-13 reduced systolic and mean blood pressure in TGA-PE rats with no effect in SD rats. Diastolic blood pressure was higher in TGA-PE versus SD rats; however, the administration of the peptide did not change diastolic blood pressures. TGA-PE had increased heart rate that was normalized by the treatment with (Pyr^1^)-apelin-13 (Fig. 2A). TGA-PE rats also had impaired heart rate variability (HRV) (measured as root of mean successive differences [rMSSD]) compared with SD controls (Fig. 2B). Apelin treatment normalized HRV in TGA-PE rats with no change in SD rats. Similarly, baroreflex sensitivity (BRS) measured in the sequence domain was lower in TGA-PE versus SD and was normalized with (Pyr^1^)-apelin-13 (Fig. 2C).

### Effects of (Pyr^1^)-apelin-13 Administration on Systemic Markers of Preeclampsia

**Figure 3:**
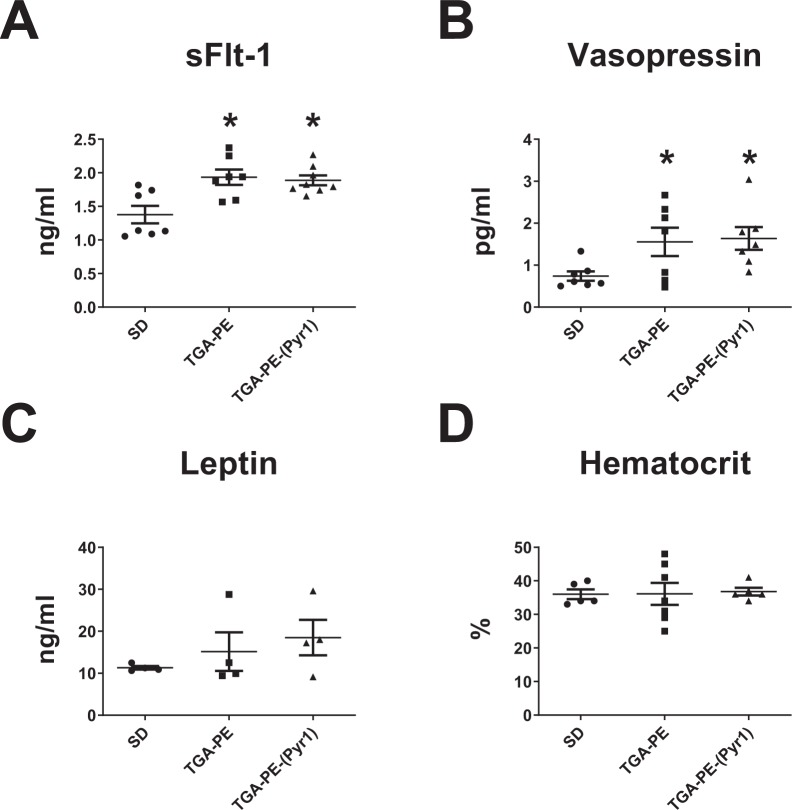
Circulating markers (sFlt-1, (**A**); Vasopressin, (**B**); Leptin, (**C**); Hematocrit, (**D**) in response to (Pyr^1^)-apelin-13 administration in TGA-PE rats at day 20 of gestation. Data are mean ± SEM. *p < 0.05 vs. SD saline-treated; n = 7–8.

Because preeclampsia is associated with higher levels of sFlt-1 and vasopressin, we tested the effects of systemic (Pyr^1^)-apelin-13 administration on these circulating markers. Plasma sFlt-1 and vasopressin were higher in TGA-PE rats versus untreated SD females at GD20. (Pyr^1^)-apelin-13 administration had no effect on these parameters (Fig. 3A,B). (Pyr^1^)-apelin-13 also had no effect on maternal serum leptin or hematocrit (Fig. 3C,D).

### Cardiac Effects of (Pyr^1^)-apelin-13

Since heart weight was greater in TGA-PE rats, we explored whether preeclampsia altered systolic function and whether (Pyr^1^)-apelin-13 had affected ejection fraction, stroke volume, cardiac output, or relative posterior wall thickness. TGA-PE rats had lower ejection fraction compared with SD rats. No significant differences in other indices of cardiac function were observed between SD and TGA-PE groups **(**Table 2**)**. (Pyr^1^)-apelin-13 increased ejection fraction in TGA-PE females. There were no differences in stroke volume, cardiac output, or relative posterior wall thickness in TGA-PE rats treated with (Pyr^1^)-apelin-13 compared with SD females (Table 2).

**Table 2:** Cardiac Function in Response to (Pyr^1^)-Apelin-13 Administration in Transgenic Rats with Preeclamptic Features (TGA-PE) at Day 20 of Gestation.

	SD	TGA-PE	TGA-PE + (Pyr^1^)
Stroke volume, ml	171.2 ± 21.5	155.9 ± 19.2	180.5 ± 20.9
Cardiac Output, ml/min	54.1 ± 7.2	48.1 ± 7.3	56.1 ± 5.5
Ejection Fraction, %	88.9 ± 2.9	79.7 ± 2.4*	90.5 ± 0.6^#^
Relative PW Thickness	0.57 ± 0.06	0.56 ± 0.04	0.73 ± 0.08

Data are mean ± SEM, *p < 0.05 vs. SD; ^#^p < 0.05 vs. TGA-PE, n = 4–5.

### Renal Effects of (Pyr^1^)-apelin-13

**Figure 4:**
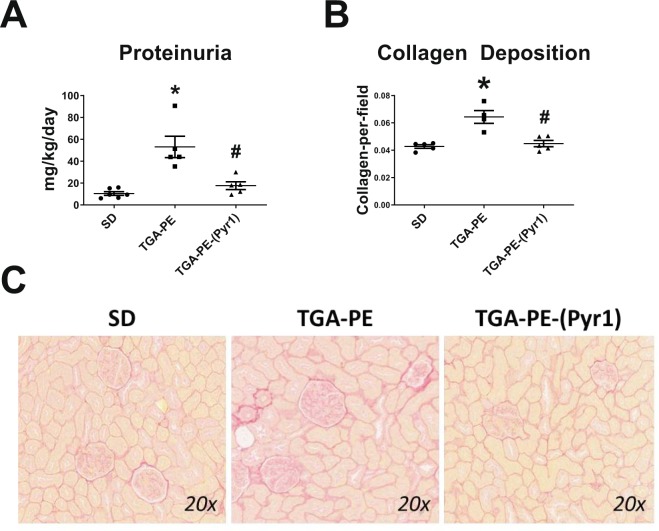
Renal proteinuria (**A**) and collagen deposition (**B**,**C**) in response to (Pyr^1^)-apelin-13 administration in TGA-PE rats at day 20 of gestation. Data are mean ± SEM. *p < 0.05 vs. SD saline-treated; ^#^p < 0.05 vs. TGA-PE saline-treated; n = 5–6.

**Figure 5:**
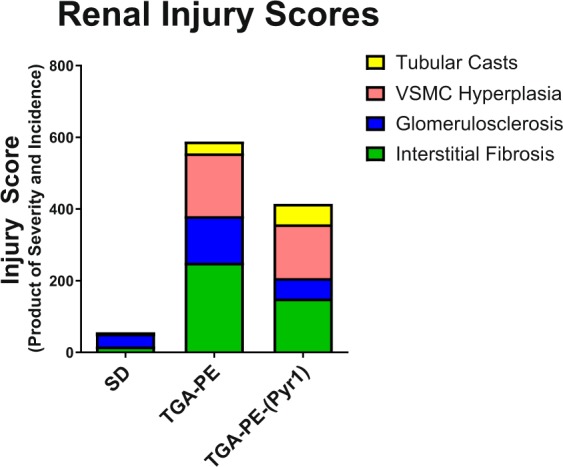
Fractional distribution of renal injury scores in SD, TGA-PE, and TGA-PE treated with (Pyr^1^)-apelin-13. Data are illustrated by treatment group.

**Figure 6:**
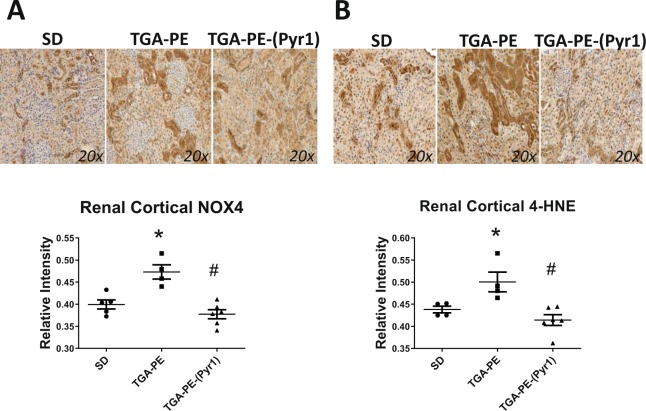
Renal NOX4 (**A**) and 4-HNE (**B**) in response to (Pyr^1^)-apelin-13 administration in TGA-PE rats at day 20 of gestation. Data are mean ± SEM. *p < 0.05 vs. SD, ^#^p < 0.05 vs. TGA-PE saline-treated; n = 4–5.

Proteinuria was greater in TGA-PE rats, and was normalized by (Pyr^1^)-apelin-13 administration (Fig. 4A). (Pyr^1^)-apelin-13 also reduced renal cortical collagen content in these rats (Fig. 4B,C). TGA-PE females had higher incidence of pathological changes of various degrees including greater injury scores for renal interstitial fibrosis, glomerulosclerosis, and VSMC hyperplasia (Table 3). (Pyr^1^)-apelin-13 administration reduced glomerulosclerosis and interstitial fibrosis, while no differences were found in injury scores for other markers between TGA-PE treated with apelin compared with TGA-PE control (Table 3). Figure 5 demonstrates fractional distribution of renal injury scores in SD, TGA-PE, and TGA-PE treated with (Pyr^1^)-apelin-13. In addition, the markers of oxidative stress NOX4 and 4-HNE were greater in TGA-PE, but were reduced following administration of (Pyr^1^)-apelin-13 in TGA-PE rats (Fig. 6).

**Table 3:** The Effect of (Pyr1)-apelin-13 Administration on Renal Pathology at Day 20 of Gestation.

	SD	SD	SD	TGA-PE	TGA-PE	TGA-PE	TGA-PE-(Pyr1)	TGA-PE-(Pyr1)	TGA-PE-(Pyr1)
Pathologic Findings	A	B	C	A	B	C	A	B	C
Interstitial Fibrosis	0.4 ± 0.2	40	16 ± 9.7	2.5 ± 0.2	100	250 ± 22.3*	1.7 ± 0.2	100	150 ± 18.3^#^
Glomerulosclerosis	0.6 ± 0.2	60	36 ± 14.7	2.1 ± 0.4	100	129.8 ± 29.7*	1.1 ± 0.1	100	57.1 ± 4.8^#^
VSMC Hyperplasia	0	0	0	1.75 ± 0.1	100	175 ± 17.1*	1.5 ± 0.2	100	150 ± 18.9*
Tubular Casts	0.2 ± 0.2	20	4 ± 4	1 ± 0.6	33.3	33.3 ± 21.1	1 ± 0.4	57.1	57.1 ± 24.9

Abbreviations: A – Severity, B – Incidence, C- Score.

SD, Sprague-Dawley, TGA-PE, preeclamptic rat, TGA-PE-(Pyr1), preeclamptic rat treated with (Pyr1)-apelin-13.

Each lesion was given a grade using a 5-point grading scale: 1 = minimal, 2 = mild, 3 = moderate, 4 = marked, 5 = severe. Injury scores are calculated as a product of a lesion’s severity grade and the incidence. The data are mean ± SEM, *p < 0.05 vs. SD, ^#^p < 0.05 vs. TGA-PE, n = 5–7.

## Discussion

As defined by the recent recommendations of the American College of Obstetricians and Gynecologists, preeclampsia is a multisystem syndrome characterized by hypertension accompanied by thrombocytopenia, impaired liver function, development of new renal insufficiency, pulmonary edema, or new-onset cerebral or visual disturbances^1^. Many advances have been made in the past decade in establishing the mechanisms of preeclampsia; however, no specific therapies are available for the treatment of this disorder. Considering the complicated nature of preeclampsia, therapeutic agents that act on multiple systems may provide the most efficacious treatment. Our study reports the therapeutic effectiveness of apelin administration in rats with preeclamptic features. We show that apelin reduces blood pressure in addition to improving parasympathetic tone, proteinuria, renal damage, and renal oxidative stress without toxic effects on maternal or fetal weight. Our findings suggest that (Pyr^1^)-apelin-13 may be beneficial for the treatment of preeclampsia due to its hemodynamic and reno-protective effects. We also report for the first time that the hypotensive effect of (Pyr^1^)-apelin-13 may involve central control of the cardiovascular system.

An established transgenic model of pregnancy with preeclamptic features was used in this study (TGA-PE). The advantages of this model are the development of many features of human preeclampsia as well as good reproducibility. Moreover, the plasma levels of apelin are lower in TGA-PE rats versus normal pregnant SD rats suggesting the downregulation of circulating apelin system. Similar to other studies using this model, blood pressure and proteinuria were significantly increased in pregnant transgenic rats^19–21^. We also report for the first time that the levels of circulating sFlt and vasopressin were greater in TGA-PE rats compared with normal pregnant rats. Since the placenta has many proteases and other protein-degrading enzymes, we chose to use a more stable to degradation form of apelin, (Pyr^1^)-apelin-13, at a dose that previously was shown to be effective in reducing blood pressure^5^.

Apelin has anti-hypertensive effects in humans and animal models when administered systemically^5,22–26^. The effectiveness of apelin’s blood pressure-lowering effects depends on several factors, including the dose, anesthesia, route of administration, and experimental model^27^. In addition, apelin receptor conformational states stabilized by the binding of different apelin fragments may also influence the hypotensive effects of apelin^26^. Our data agree with other reports^5,22–26^ showing blood pressure lowering effects of (Pyr^1^)-apelin-13 when it is administered systemically in the peripheral vascular system. Apelin can increase the levels of a major vasodilator in the circulation - nitric oxide^28^ or nitric oxide bioavailability, and therefore improve endothelial function and reduce vascular resistance^5^. Although not measured in our study, lower overall total peripheral resistance may lead to the reduction in blood pressure in response to apelin treatment.

Autonomic nervous system plays an important role in the adaptations of cardiovascular system to pregnancy. The activity of sympathetic and parasympathetic tone can be measured non-invasively using the spectral analysis method of time and frequency domains. Pregnancy lowers baroreflex sensitivity and sympathetic activity that may in part be responsible for orthostatic hypotension experienced by pregnant women^29^. Preeclampsia further impairs the baroreflex sensitivity^30^. In agreement with human studies, we report for the first time that pregnant transgenic rats develop lower parasympathetic tone at the end of pregnancy. Apelin treatment reversed this effect indicating the improvement of central nervous system regulation contributing to the blood pressure-lowering effects of apelin in preeclamptic rats. Moreover, lower baroreflex sensitivity^29^, has been linked to altered insulin sensitivity^31^. Future studies will explore links between baroreflex sensitivity, blood pressure, insulin sensitivity, and the apelin receptor system.

(Pyr^1^)-apelin-13 given from GD13 to GD20 had no effect on maternal or fetal weight, suggesting that the peptide had no overall toxicity during mid-late pregnancy. Maternal weight can determine fetal weight; therefore, the absence of changes in fetal weight may in part be explained by the absence of changes in maternal weight. In addition, no changes in food intake or leptin levels were found at the end of pregnancy in preeclamptic rats. Leptin is a hormone produced by adipose cells. Maternal circulating levels of leptin are increased in human and rat pregnancy compared with the non-pregnant state^32^. We found no differences in leptin levels in preeclamptic versus untreated SD rats and no effect of (Pyr^1^)-apelin-13 on leptin levels, suggesting no interaction between apelin and leptin in our model.

Previous studies demonstrated higher circulating levels of pro-angiogenic marker sFlt-1^33^ or anti-diuretic hormone vasopressin^34^ in women with preeclampsia compared with healthy controls. Apelin can mediate angiogenesis via upregulation of vascular endothelial growth factor (VEGF) or VEGF receptor 2^35^. Apelin can also counteract vasopressin actions by reducing plasma arginine vasopressin (AVP) levels in lactating rats or by inducing the direct effects on aquaporin 2 insertion at the apical membrane of collecting duct^36,37^. However, in our study, (Pyr^1^)-apelin-13 had no effect on sFlt-1 or vasopressin measured at the end of pregnancy in TGA-PE rats. Consistent with no changes in vasopressin with apelin treatment, urine volume was not significantly changed. Since the current view of preeclampsia emphasizes different underlying mechanisms leading to a multisystemic syndrome^38^, it is possible that apelin’s actions in our study were mediated by pathways independent of or downstream from sFlt-1 or vasopressin.

Apelin has strong inotropic effect in animal studies, including isolated heart preparations, myocardial trabeculae, and single cardiomyocytes^9,39,40^. Increased myocardial contractility partly depends on increased intracellular calcium levels or activation of inositol triphosphate receptor^40,41^. In addition, apelin administration improved cardiac contractility, and reduced infarct size in experimental models of myocardial infarction^28,42–45^. We found that heart weight was greater in TGA-PE rats compared with normal pregnant rats. To establish whether increased heart weight was accompanied with impaired cardiac function in TGA-PE rats, we determined ejection fraction. Although ejection fraction was lower in TGA-PE compared with normal pregnant rats, these values were within normal limits of this parameter in rats. A recent study reported no differences in ejection fraction in women with preeclampsia compared with women with normal pregnancy, however more detailed analysis using myocardial strain imaging with speckle tracking revealed worsened strain in preeclamptic women^46^. It is possible that TGA-PE rats have changes in the heart that can only be detected using more sensitive methods than analyzing ejection fraction. Our study showed increased systolic cardiac function in preeclamptic rats following (Pyr^1^)-apelin-13 administration as was evident by increased ejection fraction in agreement with other reports demonstrating inotropic effect of apelin. We further evaluated cardiac function in TGA-PE rats by examining cardiac output. Pregnancy increases cardiac output, stroke volume and heart rate to accommodate for higher blood volume associated with healthy pregnancy^47^. Cardiac output was not different between preeclamptic and normal pregnant rats. Although cardiac output was higher in TGA-PE treated with apelin compared with TGA-PE, it did not reach statistical significance, perhaps due to a small sample size. Higher cardiac output in addition to lower blood pressure in TGA-PE rats treated with apelin could suggest decreased total peripheral resistance. Overall, TGA-PE rats have suboptimal cardiovascular adaptations to pregnancy with preserved ejection fraction. In agreement with other studies, apelin administration positively modulated systolic cardiac function in TGA-PE rats.

Furthermore, preeclampsia can be associated with proteinuria, glomerular and tubular changes^48,49^. Apelin treatment attenuated glomerulosclerosis and interstitial fibrosis in agreement with reduced total renal collagen deposition in TGA-PE rats. These histological changes were associated with the reduction in oxidative stress markers. Lower glomerulosclerosis may in part be explained by the improvement of renal hemodynamics since apelin had been shown to induce vasorelaxation of angiotensin II-preconstricted efferent and afferent arterioles^36^. However, apelin did not have a significant effect on VSMC hyperplasia or tubular casts in TGA-PE rats. Short-term treatment with apelin may not be enough to reverse all damage in the kidney and other mechanisms that were not influenced by apelin could be responsible for vascular damage in the kidney of rats with preeclamptic features. Further studies are warranted on investigating the mechanisms of apelin actions in the kidney in preeclampsia. Overall, apelin improved renal pathology in addition to lowering oxidative stress markers, suggesting that (Pyr^1^)-apelin-13 may provide reno-protection in preeclampsia.

### Perspectives

Preeclampsia is a complex multifactorial syndrome that currently has no cure. Our study opens the possibility of considering apelin as an important mediator of hemodynamic and renal pathophysiology in preeclampsia due to its blood pressure-lowering and reno-protective effects. Future studies are needed to investigate the tissue-specific mechanisms of apelin’s actions in preeclampsia.

## Materials and Methods

### Animals

The study was approved by the Institutional Animal Care and Use Committee of the Wake Forest School of Medicine (A14-163), and all experiments and methods were performed in accordance with relevant guidelines and regulations. Female transgenic rats with overexpression of human angiotensinogen (TGA) or male transgenic rats with overexpression of human renin (TGR) were obtained from the colony maintained by the Department of Surgery/Hypertension and Vascular Research, Wake Forest School of Medicine. Sprague-Dawley (SD) rats purchased from Charles River Laboratories (Wilmington, MA, USA) were used in control experiments. All rats were housed at a constant room temperature, humidity, and light cycle (12:12-h light-dark), fed a standard rodent chow (Lab Diet 5P00 - Prolab RMH 3000, PMI Nutrition International, INC, Brentwood, MO) and given water *ad libitum* throughout the experimental protocols.

### Study timeline

Female TGA rats were mated to male TGR rats to develop preeclamptic features (TGA-PE). SD females were bred to SD male rats. First time pregnant TGA-PE and SD rats at 10–15 weeks of age were used for this study. Day 0 of pregnancy was established by the presence of a vaginal plug or sperm in the vaginal smear. (Pyr^1^)-apelin-13 (2 mg/kg/day)^5^ (American Peptide, Sunnyvale, VA) or saline was infused subcutaneously in TGA-PE or SD female rats via osmotic minipumps (model 2ML4; Alzet Osmotic Pumps, Cupertino, CA) starting at day 13 of gestation (GD13). Cardiac echocardiography was done in all rats at GD19 using a Vevo LAZR Ultrasound and Photoacoustic System (FujiFilm, VisualSonics, Toronto, Canada). On the evening of GD19, animals were placed in metabolic cages for 24-hour collection of urine (MMC 100, Hatteras Instruments, Cary, NC). Blood pressures were taken in the morning of the following day and rats were sacrificed in the afternoon of GD20.

### Blood pressure recordings

Systolic, diastolic, and mean blood pressures were recorded using an intra-arterial catheter placed into the left femoral artery under 2.5% isoflurane anesthesia. Blood pressure signals were acquired for 10 minutes using PowerLab equipment (ADInstruments, Colorado Springs, CO) and analyzed by LabChart software. Heart rate was also calculated from this recording as beats per minute. Data were averaged for each animal and reported as mean ± SEM.

### Heart rate variability and baroreflex measurements

Indices of sympathovagal activity were calculated by spectral analysis of the time and frequency domains using software designed for rats (Nevrokard SA‐BRS, Nevrokard Kiauta, Izola, Slovenia), as previously described^50,51^. Spontaneous baroreflex sensitivity (BRS) was determined from a minimum of 10 min of arterial blood pressure recordings and was calculated in the time domain as Sequence ALL (Seq ALL; in units of milliseconds per mmHg). Time‐domain analysis was used to calculate measures of heart rate variability (HRV), an index of cardiac vagal tone, measured in milliseconds as root of mean square of successive differences (rMSSD).

### Ultrasound measurements

Animals were placed on a temperature controlled platform. Chest hair was removed using a depilatory cream (Nair, Church & Dwight Co., Inc). Echocardiography was performed using a Vevo LAZR Ultrasound and Photoacoustic System and MS250S transducer under 1.5% isoflurane anesthesia. Heart rate was determined from 5 consecutive RR intervals using ECG electrodes connected to a temperature controlled platform. Left ventricle M-mode images were recorded in the short-axis parasternal view. The ejection fraction (EF) was calculated as %EF = ((left ventricle end-diastolic volume (LVEDV) − left ventricular end-systolic volume (LVESV)/LVEDV) × 100. The relative wall thickness was calculated as (posterior wall thickness in diastole (PWTd) + anterior wall thickness in diastole (AWTd)/left ventricular end-diastolic diameter (LVEDD). Stroke volume (SV) was determined as SV = (EDV − ESV) µl. Cardiac output (CO) was determined as cardiac output (CO) = heart rate (HR) × SV/1000 ml/min.

### Enzyme immunoassay (EIA) for circulating apelin

Apelin-12 extraction free Enzyme immunoassay kit (EIA), protocol 2 (Phoenix Pharmaceuticals, Inc., Burlingame, CA) was used to measure serum levels of apelin in SD and TGA-PE rats. This assay recognizes all forms of apelin and measures total apelin levels.

### Sirius Red collagen staining

Paraffin-embedded kidney sections (5μm) were stained with standard Sirius Red staining to identify collagen content using reagents from Sigma-Aldrich (St. Louis, MO). Sections were incubated in 0.1% Picro-Sirius Red solution followed by 0.5% Glacial Acetic acid. Slides were scanned with a Hamamatsu Nanozoomer 2.0 HT utilizing NDP.scan and NDP.view as the imaging software at 20X magnification (Hamamatsu). The total area of collagen deposition (red staining) was identified in 12 sections of the digitized images from each kidney (n = 6–7) and measured as the collagen-to-field ratio using Adobe Photoshop 7.0. Analyses were performed by an investigator blinded to the experimental groups.

### Injury scores were determined using Sirius Red Collagen staining

The following criteria were used to identify renal injury as previously described by us^52,53^: for glomerulosclerosis - increased collagen deposition within the glomerular or periglomerular area; for vascular smooth muscle cell hyperplasia – changes in the cellularity/organization of layers observed in arteries; for interstitial fibrosis - an increased amount of collagen between tubules; greater degrees of fibrosis were graded higher if more parenchyma was involved; for tubular casts – presence of cast within the tubule. Each lesion was given a grade using a 5-point grading scale: grade 1 = minimal, grade 2 = mild, grade 3 = moderate, grade 4 = marked, grade 5 = severe. The data are reported as injury scores calculated as a product of a lesion’s severity grade and the incidence.

### Immunohistochemistry

Kidney tissues were fixed in formalin and 70% ethanol, embedded in paraffin and cut into 5-µm sections. Immunostaining was performed using the avidin biotin complex (ABC) method with 0.1% diaminobenzene solution used as the chromogen as previously described^52,53^. Antigen retrieval treatment with sodium citrate buffer (pH 6.0) was applied at 90–95 °C for 30 min. Non-specific binding was blocked in a buffer containing 10% normal goat serum, 1% Triton-X in PBS for 30 min. Kidney sections were incubated with the rabbit polyclonal 4-hydroxy-2-nonenal Michael Adducts (4-HNE; dilution: 1:10,000; Calbiochem, La Jolla, CA), rabbit polyclonal NOX-4 antibody (dilution: 1:200; Proteintech, Rosemont, IL, USA) and secondary biotinylated goat anti-rabbit antibody (dilution: 1:400; Vector Laboratories, Burlingame, CA, USA). Slides were scanned with a Hamamatsu Nanozoomer 2.0 HT utilizing NDP.scan and NDP.view as the imaging software at 0.25 and 20X magnification. Images of 4-HNE or NOX-4 staining were analyzed using Adobe Photoshop 7.0 in 9 sections from each kidney cortex (n = 4–5) by an investigator blinded to the experimental groups and the data are reported as relative intensity units.

### Statistics

All data were tested for normality. Groups were compared using two-way analysis of variance (ANOVA) or one-way ANOVA followed by the Bonferroni’s post-tests (GraphPad Software, San Diego, CA). A p value less than 0.05 was considered statistically significantly different. All data were presented as mean ± SEM.

## Data Availability

Data is available from the authors upon reasonable request.

## References

[1] Roberts JM (2013). Hypertension in Pregnancy: Executive Summary. Report of the American College of Obstetricians and Gynecologists’ Task Force on Hypertension in Pregnancy. Obstet Gynecol.

[2] Davis EF (2012). Pre-eclampsia and offspring cardiovascular health: mechanistic insights from experimental studies. Clinical Science (London, England: 1979).

[3] Ananth CV, Keyes KM, Wapner RJ (2013). Pre-eclampsia rates in the United States, 1980-2010: age-period-cohort analysis. The BMJ.

[4] Wallis AB, Saftlas AF, Hsia J, Atrash HK (2008). Secular Trends in the Rates of Preeclampsia, Eclampsia, and Gestational Hypertension, United States, 1987–2004. American Journal of Hypertension.

[5] Chun HJ (2008). Apelin signaling antagonizes Ang II effects in mouse models of atherosclerosis. J Clin Invest.

[6] Ishida J (2004). Regulatory roles for APJ, a seven-transmembrane receptor related to angiotensin-type 1 receptor in blood pressure *in vivo*. J Biol Chem.

[7] Tatemoto K (1998). Isolation and characterization of a novel endogenous peptide ligand for the human APJ receptor. Biochem Biophys Res Commun.

[8] Zhong JC (2007). Apelin modulates aortic vascular tone via endothelial nitric oxide synthase phosphorylation pathway in diabetic mice. Cardiovasc Res.

[9] Szokodi I (2002). Apelin, the novel endogenous ligand of the orphan receptor APJ, regulates cardiac contractility. Circ Res.

[10] Cox CM, D’Agostino SL, Miller MK, Heimark RL, Krieg PA (2006). Apelin, the ligand for the endothelial G-protein-coupled receptor, APJ, is a potent angiogenic factor required for normal vascular development of the frog embryo. Dev Biol.

[11] Kasai A (2008). Retardation of retinal vascular development in apelin-deficient mice. Arterioscler Thromb Vasc Biol.

[12] Yamaleyeva LM (2015). Downregulation of apelin in the human placental chorionic villi from preeclamptic pregnancies. Am J Physiol Endocrinol Metab.

[13] Furuya M (2012). Expression of angiotensin II receptor-like 1 in the placentas of pregnancy-induced hypertension. Int J Gynecol Pathol.

[14] Hosoya M (2000). Molecular and functional characteristics of APJ. Tissue distribution of mRNA and interaction with the endogenous ligand apelin. J Biol Chem.

[15] Bortoff KD, Qiu C, Runyon S, Williams MA, Maitra R (2012). Decreased maternal plasma apelin concentrations in preeclampsia. Hypertens Pregnancy.

[16] Kucur M (2014). Maternal serum apelin and YKL-40 levels in early and late-onset pre-eclampsia. Hypertens Pregnancy.

[17] Inuzuka H (2013). Decreased expression of apelin in placentas from severe pre-eclampsia patients. Hypertension in Pregnancy.

[18] Malamitsi-Puchner A (2007). Circulating apelin concentrations in mother/infant pairs at term. Acta Paediatr.

[19] Hering L (2010). Effects of circulating and local uteroplacental angiotensin II in rat pregnancy. Hypertension.

[20] Herse F (2007). Dysregulation of the circulating and tissue-based renin-angiotensin system in preeclampsia. Hypertension.

[21] Herse F (2008). AT1-receptor autoantibodies and uteroplacental RAS in pregnancy and pre-eclampsia. J Mol Med (Berl).

[22] Akcilar R (2013). Apelin effects on blood pressure and RAS in DOCA-salt-induced hypertensive rats. Clin Exp Hypertens.

[23] Barnes GD (2013). Sustained cardiovascular actions of APJ agonism during renin-angiotensin system activation and in patients with heart failure. Circ Heart Fail.

[24] Japp AG (2010). Acute cardiovascular effects of apelin in humans: potential role in patients with chronic heart failure. Circulation.

[25] Lee DK (2000). Characterization of apelin, the ligand for the APJ receptor. J Neurochem.

[26] Lee DK (2005). Modification of the terminal residue of apelin-13 antagonizes its hypotensive action. Endocrinology.

[27] Yamaleyeva LM, Shaltout HA, Varagic J (2016). Apelin-13 in blood pressure regulation and cardiovascular disease. Curr Opin Nephrol Hypertens.

[28] Azizi Y, Faghihi M, Imani A, Roghani M, Nazari A (2013). Post-infarct treatment with [Pyr1]-apelin-13 reduces myocardial damage through reduction of oxidative injury and nitric oxide enhancement in the rat model of myocardial infarction. Peptides.

[29] Brooks VL, Cassaglia PA, Zhao D, Goldman RK (2012). Baroreflex function in females: changes with the reproductive cycle and pregnancy. Gend Med.

[30] Molino P (1999). Baroreflex control of heart rate is impaired in pre-eclampsia. J Hum Hypertens.

[31] Zhao D, McCully BH, Brooks VL (2012). Rosiglitazone improves insulin sensitivity and baroreflex gain in rats with diet-induced obesity. J Pharmacol Exp Ther.

[32] Henson MC, Castracane VD (2000). Leptin in Pregnancy. Biology of Reproduction.

[33] Zeisler H (2016). Predictive Value of the sFlt-1:PlGF Ratio in Women with Suspected Preeclampsia. N Engl J Med.

[34] Santillan MK (2014). Vasopressin in preeclampsia: a novel very early human pregnancy biomarker and clinically relevant mouse model. Hypertension.

[35] Zeng H, He X, Hou X, Li L, Chen JX (2014). Apelin gene therapy increases myocardial vascular density and ameliorates diabetic cardiomyopathy via upregulation of sirtuin 3. Am J Physiol Heart Circ Physiol.

[36] Hus-Citharel A (2008). Effect of apelin on glomerular hemodynamic function in the rat kidney. Kidney International.

[37] Hus-Citharel A (2014). Apelin counteracts vasopressin-induced water reabsorption via cross talk between apelin and vasopressin receptor signaling pathways in the rat collecting duct. Endocrinology.

[38] Maric-Bilkan C (2019). Research Recommendations From the National Institutes of Health Workshop on Predicting, Preventing, and Treating Preeclampsia. Hypertension.

[39] Perjes A (2014). Apelin increases cardiac contractility via protein kinase Cepsilon- and extracellular signal-regulated kinase-dependent mechanisms. PLoS One.

[40] Wang C, Du JF, Wu F, Wang HC (2008). Apelin decreases the SR Ca2+ content but enhances the amplitude of [Ca2+]i transient and contractions during twitches in isolated rat cardiac myocytes. Am J Physiol Heart Circ Physiol.

[41] Farkasfalvi K (2007). Direct effects of apelin on cardiomyocyte contractility and electrophysiology. Biochem Biophys Res Commun.

[42] Azizi Y (2015). Post-infarct treatment with [Pyr(1)]apelin-13 improves myocardial function by increasing neovascularization and overexpression of angiogenic growth factors in rats. Eur J Pharmacol.

[43] Hou X, Zeng H, He X, Chen JX (2015). Sirt3 is essential for apelin-induced angiogenesis in post-myocardial infarction of diabetes. J Cell Mol Med.

[44] Li L, Zeng H, Chen JX (2012). Apelin-13 increases myocardial progenitor cells and improves repair postmyocardial infarction. Am J Physiol Heart Circ Physiol.

[45] Li L, Zeng H, Hou X, He X, Chen JX (2013). Myocardial injection of apelin-overexpressing bone marrow cells improves cardiac repair via upregulation of Sirt3 after myocardial infarction. PLoS One.

[46] Shahul S (2012). Subclinical left ventricular dysfunction in preeclamptic women with preserved left ventricular ejection fraction: a 2D speckle-tracking imaging study. Circ Cardiovasc Imaging.

[47] Nicolaides KH, Kametas NA, Bamfo JEAK, Chambers JB (2007). Maternal left ventricular diastolic and systolic long-axis function during normal pregnancy. European Journal of Echocardiography.

[48] Gaber LW, Spargo BH, Lindheimer MD (1994). Renal pathology in pre-eclampsia. Baillieres Clin Obstet Gynaecol.

[49] Codsi E (2017). Longitudinal characterization of renal proximal tubular markers in normotensive and preeclamptic pregnancies. Am J Physiol Regul Integr Comp Physiol.

[50] Shaltout HA, Abdel-Rahman AA (2005). Mechanism of fatty acids induced suppression of cardiovascular reflexes in rats. J Pharmacol Exp Ther.

[51] Shaltout HA (2010). Acute AT(1)-receptor blockade reverses the hemodynamic and baroreflex impairment in adult sheep exposed to antenatal betamethasone. Am J Physiol Heart Circ Physiol.

[52] Yamaleyeva LM (2012). Amelioration of renal injury and oxidative stress by the nNOS inhibitor L-VNIO in the salt-sensitive mRen2.Lewis congenic rat. J Cardiovasc Pharmacol.

[53] Yamaleyeva LM, Gallagher PE, Vinsant S, Chappell MC (2007). Discoordinate regulation of renal nitric oxide synthase isoforms in ovariectomized mRen2. Lewis rats. Am J Physiol Regul Integr Comp Physiol.

